# Conspiracy Theories: A Public Health Concern and How to Address It

**DOI:** 10.3389/fpsyg.2021.682931

**Published:** 2021-07-28

**Authors:** Marie-Jeanne Leonard, Frederick L. Philippe

**Affiliations:** Department of Psychology, Université du Québec à Montréal, Montreal, QC, Canada

**Keywords:** conspiracy theories, public health, self-determination theory, needs, radicalization, conspiracy beliefs

## Abstract

The SARS-CoV-2 pandemic was characterized by a significant increase in the endorsement of conspiracy theories. Conspiracy theories are narratives that can enable and accentuate distrust toward health professionals and authorities. As such, they can lead to violent radicalization and should be considered a public health issue. This perspective article aims to further the understanding of professionals on conspiracy theories *via* the 3N model of radicalization and self-determination theory. Based on empirical research, theory, and existing interventions, potential initiatives intended to tackle the issue of conspiracy theories during pandemics are also presented.

## Introduction

Increasingly, people around the globe grow tired and frustrated in response to lockdowns and health measures that are meant to counter the ongoing SARS-CoV-2 pandemic. This angst is partly manifested through the increased popularity of conspiracy theories (European Commission, 2020). An international survey carried out in 28 countries revealed that one-third of people worldwide believe that “a foreign power/other force” consciously caused the current pandemic (e.g., 18, 58, and 26% believe so in the United Kingdom, Bulgaria, and Thailand, respectively; Gallup International Association, 2020). Such conspiracy beliefs represent public health issues contributing to the fracture of the trust civilians hold toward government officials and health professionals, a phenomenon that has been observed during other disease outbreaks (Cohn and Kutalek, 2016). The objective of this perspective article is to improve the understanding of conspiracy theories, to discuss how they impact the population, and to highlight potential ways to intervene during pandemics based on theory, empirical research, and existing interventions.

We argue that conspiracy theories should be considered as narratives that can lead to violent radicalization and, as such, this phenomenon represents an important public health issue. Conspiracy theories are better understood *via* the 3N model of radicalization (Kruglanski et al., 2019) and self-determination theory (Ryan and Deci, 2017). The 3N model specifies three pillars in the radicalization process that align with the understanding of conspiracy theories (i.e., *Need, Narrative,* and *Network*), while self-determination theory deepens the understanding of the *Need* pillar.

## Conspiracy Theories

*Need* refers to the motivation to recover significance following its loss due to an adverse event (Kruglanski et al., 2019). Specifically, this significance loss can be conceptualized as the thwarting of three psychological needs that were found to be universal: competence, autonomy, and relatedness (Ryan and Deci, 2017). Indeed, the satisfaction or frustration of these three psychological needs influences how people perceive and react to an event (Ryan and Deci, 2017). The satisfaction of these psychological needs is considered as a continual quest, and when they are not satisfied or are frustrated, people naturally seek to fulfill them (Sheldon and Gunz, 2009; Ryan and Deci, 2017). They are, therefore, considered as core determinants of motivation that lead one to act on their environment and to carry certain objectives (Sheldon and Gunz, 2009; Ryan and Deci, 2017). Conspiracy theories unfold following an important event that hinders the perception of control of an individual (autonomy), the ability of an individual to make sense of the world (competence), and connectedness of an individual (relatedness; Ryan and Deci, 2017; van Prooijen, 2020). In 2020, many countries enforced a lockdown for months, a significant event that precipitated economic uncertainty and restrained individual freedom. Many have perceived their basic needs as thwarted as they lost control over their usual occupations, they were cut off from their loved ones, and authorities disseminated mixed messages because they did not (and still do not) fully understand the new virus. Such stressful events are likely to reactivate the recall of past personal life events that were need thwarting in a similar fashion (e.g., experiences of ostracism, natural disasters, or other traumas), thus exacerbating the current need thwarting experience of the pandemic (Philippe and Houle, 2020). A vulnerability to the reactivation of such need thwarting memories can motivate one to retrieve significance by finding compensatory ways to fulfill those needs, making one cognitively receptive to new narratives.

Conspiracy theories are a form of *Narrative*, defined as a shared belief system providing an alternative ideological framework to explain a situation and offer guidance as to how to behave to regain significance, control, and affiliations (Kruglanski et al., 2019). As conspiracy theories are alternative narratives to the *status quo*, people turn to them to compensate for their thwarted needs. Such narratives accentuate the threatening characteristics of demonized outgroups and praise the people who endorse the beliefs and behaviors put forward by the ideology, hence cognitively and behaviorally polarizing believers (Kruglanski et al., 2019). Of note, some authors have suggested that believing in conspiracy theories can be adaptive to survival (van Prooijen, 2020); however, one could argue that such beliefs could be considered adaptive only when they concern real conspiracies and not unjustified conspiracy theories. In the past, some conspiracies were proved to be real, rendering it difficult to distinguish unjustified conspiracy theories from real conspiracies. Unjustified conspiracy theories are not adaptive for survival, as they can be detrimental to the health of others and the individual (e.g., SARS-CoV-2 being a hoax, vaccines being dangerous, etc.). The European Commission (2020) characterizes real conspiracies as focusing on a specific event or individual, such as an assassination, and based on valid proof and facts brought forward by “whistle-blowers and the media.” The European Commission (2020) adds that whistle-blowers recognize the extent and limitations of their knowledge instead of being rigid and absolute about it. On the other hand, unjustified conspiracy theories are characterized by five distinctive elements: pattern, agency, coalition, threat, and secrecy (van Prooijen and van Vugt, 2018). Such narratives are not parsimonious and detail a pattern establishing causal relationships between multiple events, people, and/or objects (van Prooijen and van Vugt, 2018). These narratives also imply agency, that is, they attribute intentions to malevolent and malicious groups of people who form a coalition and who have a deliberate plan that represents a threat to the ingroup (van Prooijen and van Vugt, 2018). Above all else, these theories are characterized by a secrecy element, which makes them irrefutable.

When one adheres to a narrative, they then seek the presence of like-minded individuals, forming a *Network* (Kruglanski et al., 2019). In the past few months, those who endorsed a conspiracy theory on SARS-CoV-2 connected *via* social media, creating echo chambers. These echo chambers spread the reinforcement of both individual and collective actions that exacerbated tensions between civilians and impeded the initiatives of authorities to halt the propagation of the virus around the globe, propelling actions such as civil disobedience, maskless demonstrations, or a refusal to get tested and vaccinated.

Conspiracy theories and their associated actions are endorsed to satisfy the thwarted needs and fulfill the lost sense of significance. However, conspiracy theories are not expected to truly satisfy the basic needs, they potentially, only temporarily, compensate for them. Research has indeed found that when needs are experienced as frustrated, they lead the individual to rigidly engage in activities that appear to overcome some of the insecurities and threats stemming from need frustration, but this engagement falls short of achieving this and often leads to worst consequences (for a review, refer to Vansteenkiste et al., 2020). Empirical data suggest that conspiracy theories may cultivate the thwarting of the basic needs, creating a feedback loop in which the person is further reinforced to support and expand their beliefs in conspiracy theories (Douglas et al., 2017; van Prooijen, 2020). For instance, it has been shown that exposure to conspiracy theories decreases the feelings of control and autonomy by increasing perceptions of powerlessness (Jolley and Douglas, 2014a,b; Douglas et al., 2017). This is not surprising given that, although conspiracy theories offer an alternative explanation to adverse events, they emphasize how the actions of individuals cannot have an impact on the *status quo*, possibly further thwarting the need for autonomy. They also highlight how numerous events, people, and objects are interrelated, as well as how the truth is hidden from the public, likely frustrating the sense of competence of individuals. Eventually, conspiracy theories can increase the feeling of being socially alienated, as they underscore how the ingroup is badly treated by the outgroup and how the ingroup is the target of threatening plans. In addition, conspiracy theories are often a source of interpersonal conflicts between conspiracy believers and non-believers, possibly negatively affecting relationships and friendships and further reinforcing the frustration of the need for relatedness.

## Past Health Crises and Conspiracy Theories

Past health crises provide some insights into the development of conspiracy theories concerning viruses. The element of mistrust toward mainstream media, influential figures, authorities, and/or governmental institutions was the most recurring theme reported to facilitate the endorsement and spread of conspiracy theories throughout past epidemics as various as the Zika (Kou et al., 2017; Smallman, 2018), Ebola (Masumbuko Claude et al., 2019; Vinck et al., 2019), or H1N1 (Smallman, 2015). Many of these virus-related conspiracies revolved around capitalistic ill-intentions among powerful individuals (Smallman, 2015, 2018; Masumbuko Claude et al., 2019).

Trust can be modulated by real conspiracies unveiled in the past, past persecutions, and disparities perpetrated by authority figures toward minority groups (Smallman, 2015, 2018; Abramowitz et al., 2017; Kou et al., 2017) or by how authorities and institutions are perceived to competently manage an epidemic (Masumbuko Claude et al., 2019). In addition, we argue that mistrust toward institutions and the government could be a pre-epidemic social symptom engendered by past experiences of need frustration (e.g., social ostracism and injustice), which could be triggered or exacerbated by a health crisis and subjectively experienced as mistrust due to the loss of significance. This mistrust could then be further affected by how authorities and policymakers alienate the psychological needs of the population through their management of a health crisis and the enforcement of sociosanitary measures. This lack of trust is to be taken seriously, as it has been associated with a higher risk of not complying with measures and behaviors intended to contain the spread of a virus (e.g., refusing to get vaccinated, avoiding to consult with a health professional, etc. Vinck et al., 2019).

## A Public Health Concern

There is a dire need for official authorities worldwide to tackle this issue, as conspiracy endorsement feeds at least three clear public health concerns. First, the actions perpetrated by conspiracy believers increase the risk of contracting and propagating SARS-CoV-2 among the population, which, unsurprisingly, puts resources and financial pressures on health care systems. In addition, conspiracy theories can have economic repercussions at an individual level. Individuals of low socioeconomic status can see their financial struggles increase due to influential conspiracy leaders who tend to ask for financial donations to fund their organizations and to continue promoting their alternative narratives. Financial donations are often portrayed by conspiracy narratives as a way to restore the lost sense of significance. Last, people integrating conspiracy theories as core beliefs should be of high concern for governmental representatives and health professionals. Such core beliefs increase the likelihood of one endorsing other conspiracy theories and networks in the long run (van Prooijen, 2020), potentially further bolstering their distrust and rejection of traditional medicine (e.g., vaccines, treatments, and basic hygienic measures).

## Potential Initiatives

Policymakers and authorities should be careful to not circulate mixed and confusing messages at a given time (Abramowitz et al., 2017), as past epidemics were marked by the dissemination of ambivalent messages on the virus at play (Kou et al., 2017). However, changing information is not necessarily synonymous with mixed messages (Carlsen and Glenton, 2016). To prevent their information and messages from being considered as mixed, policymakers and authorities should “acknowledge” the presence of uncertainty and that the information disseminated will be adjusted as time goes by (Carlsen and Glenton, 2016). Otherwise, people might consider and interpret mainstream information as misinformation (Ball and Maxmen, 2020). Furthermore, Ball and Maxmen (2020) emphasized that authorities and policymakers should describe the reasons and rationale that “guide” the changed decisions during an epidemic.

We recommend the following societal and individual-level initiatives to defuse conspiracy beliefs and prevent them from cognitively crystalizing in the long run. These initiatives are conceived from empirical studies, professional reports, and successful measures involved in the adequate handling of past epidemics (Niang, 2015; Cohn and Kutalek, 2016; Baggio et al., 2019; Sigfrid et al., 2020). Studies have suggested that increasing the sense of control, perception of transparency, and self-affirmation (i.e., asserting the values, meaning, and feelings of an individual) can decrease the strength of conspiracy beliefs (Whitson and Galinski, 2008; Carlsen and Glenton, 2016; Douglas et al., 2017; Poon et al., 2020; van Prooijen, 2020). There is a consensus among professionals that engaging the population and genuinely listening to their needs, perceptions, and concerns greatly helps to ensure the efficacy of sanitary measures during disease outbreaks (Niang, 2015; Cohn and Kutalek, 2016; Baggio et al., 2019). Indeed, public health measures to contain past Ebola epidemics were varied and included community engagement initiatives (Coltart et al., 2017). Though Coltart et al. (2017) consider it unclear which initiatives worked best, as they were usually implemented at the same time, the authors highlighted that community engagement initiatives seem to act as a catalyst to other initiatives (Coltart et al., 2017). Community engagement measures potentially work by increasing trust toward authorities, therefore increasing compliance and adequate function of other public health measures (Coltart et al., 2017).

Therefore, we first strongly advise state and local governments to invest in the implementation of a “qualitative community feedback mechanism” as was implemented for instance by the Democratic Republic of Congo as an additional measure for Ebola epidemics (Baggio et al., 2019; Nachega et al., 2020). This mechanism collected perceptions and comments of people about the epidemic (i.e., Ebola) to (1) improve trust and engagement of communities and (2) help officials with feedback to adapt their decisions and priorities in terms of the needs of the community (Baggio et al., 2019). The information collected by the feedback mechanism was afterward coded, analyzed, and shared with the adequate authorities (Baggio et al., 2019). Such a mechanism allows each citizen to feel part of the larger social network and favors community engagement and empowerment, elements identified as key targets during an epidemic to reduce fears and rumors (Cohn and Kutalek, 2016; Baggio et al., 2019; Sigfrid et al., 2020). Therefore, state and local governments should ensure their population the universal access to a feedback mechanism regarding SARS-CoV-2 so that all can demonstrate self-affirmation by expressing their thoughts, perceptions, and questions about the current pandemic (Poon et al., 2020; van Prooijen, 2020). Special advisors should be appointed to oversee the mechanism and the adequate communication between involved parties (Sigfrid et al., 2020). State and local governments should also take the time to publicly and transparently respond to questions and comments raised *via* the feedback mechanism (including recognizing the limitations of government in understanding SARS-CoV-2), thus demonstrating that concerns of civilians are considered in the decision-making process (Niang, 2015; Baggio et al., 2019). The aim should not simply be to convince people of the “truth” but rather to create a bidirectional communication system allowing a true connection of trust between civilians and officials (O’Malley et al., 2009) and acknowledge the emotional stress induced by the current situation. This type of mechanism would support the needs for autonomy, competence, and relatedness and would therefore potentially reconnect one with truthful information regarding SARS-CoV-2 (Goldberg and Richley, 2020).

Second, at a micro-level, when health professionals face a person who endorses a conspiracy theory, we strongly recommend them to open a non-judgmental dialogue and instill a sense of trust in the relationship. Given the premise that endorsed conspiracy beliefs are a failed attempt to satisfy thwarted needs, these dialogues represent an opportunity that must be grasped (1) to let the person express their beliefs in a safe environment, (2) to respectfully offer rational counter-arguments if necessary, (3) to rationally explore why the counter-arguments are frustrating and how the conspiracy beliefs could keep one frustrated in terms of autonomy, competence, and/or relatedness, and (4) to investigate healthy ways to fulfill the needs so that the person can reconnect with others (Niang, 2015; Orosz et al., 2016). Such actions have the potential to satisfy the initially thwarted needs, therefore curbing the influence of conspiracy beliefs.

Finally, not all believers in conspiracy theories are on the track to radicalization. Kou et al. (2017) described how “in the face of crisis, people face enormous uncertainty and have urgent information needs,” which translates into important needs for autonomy and competence. Some, in a quest for “collective sensemaking,” (Kou et al., 2017) were only deceived by fake news advertised on social media or were misinformed by a trusted close other (European Commission, 2020). Around the world, some countries tried to counter distrust regarding SARS-CoV-2 by employing fear campaigns that advertised threatening health messages, further frustrating the need for competence and autonomy (Stolow et al., 2020). Research suggests that these campaigns are ineffective, as they are experienced as controlling and only extrinsically motivate one to comply with the recommended health measures (Williams et al., 1999). They often have a short-term modest effect (Tannenbaum et al., 2015), mostly among people who are already in agreement with the message, but they fail to change the behaviors of the target group for which they were developed (Ruiter et al., 2014). Instead, authorities should employ rumor detectors (Baggio et al., 2019) specifically targeting fake news regarding the pandemic^1^. Such a detector would have the potential to provide tailored information based on the misunderstandings of the population and unfounded rumors not reported *via* the qualitative community feedback mechanism. Based on this detector, authorities could make a weekly review and disseminate it to the media (e.g., editorials and radio stations) to inform the general population and disseminate it to elementary, high school, and college professors to ensure the circulation of adequate and truthful information among youth.

## Getting Policymakers to Implement Changes

At the moment, there is a need to raise awareness of policymakers on the issue of conspiracy endorsement. To do so, we strongly encourage researchers and other professionals working in the field of conspiracy to increase their presence in the media (e.g., *via* radio hosts and journalists) to circulate reliable information and to get the conversation going on the issue of conspiracy endorsement (Burstein, 2003). By raising the salience of the issue and by changing the public opinion on this issue, the chances of getting the attention of policymakers increase (Burstein, 2003). In addition, more articles and materials written in lay language are needed (Economic and Social Research Council, 2021). Such articles and material could be tailored for publishing in daily newspapers known to be read by policymakers (e.g., The Hill Times in Ottawa, Canada). The members of the public who feel concerned by the issue of conspiracy endorsement should be encouraged to lobby and/or to solicitate their local or state government representatives *via* emails, phone calls, and in-person meetings to encourage the implementation of preventive initiatives. The civic engagement action of lobbying could even increase the satisfaction of the three psychological needs by rallying members of the public with an objective, providing a sense of self-efficacy, and producing a sense of control within their society.

To increase awareness and highlight the scale of the issue of conspiracy endorsement in the population, we further propose that researchers investigate the societal costs generated by conspiracy endorsement and their associated behaviors. Such societal costs would include the direct costs related to mental health issues and the actions associated with endorsed conspiracy theories, including health services used due to mental and/or physical health issues and their indirect impact on loss of productivity at work and job turnover. The direct costs could be two-fold; on the one hand, people who endorse conspiracy theories are at risk of refusing and avoiding traditional health care services (including refusing vaccines) in favor of alternative medicines, which could have long-term impacts on their health and the spread of viruses in the general population. On the other hand, the development of long-term diseases and/or the development of mental health issues could have a direct economic impact on health care services used (e.g., emergency room visits, hospitalizations, psychiatric hospitalization, and medication use).

The understanding of health professionals on conspiracy theories should also be actualized and updated. Professional orders (i.e., doctors, psychologists, and socials workers) should encourage health professionals to attend continuous training activities demystifying conspiracy theories and how to address them when confronted with an individual endorsing them.

## Accountability and Transparency

Given the importance of the issue of mistrust toward authorities, the handling of actions perpetrated by governments and health professionals that can reinforce conspiracy theories is to be considered. Indeed, past conspiracies that were revealed as true have served as fuel for the current unjustified conspiracy theories circulating (e.g., Tuskegee experiment, Wikileaks, etc.; Smallman, 2018; Pierre, 2020). To facilitate the implementation of the proposed initiatives and increase trust, authorities and policymakers should support and value the concepts of accountability and transparency (Coltart et al., 2017). Accountability is important to value due to the secrecy element characterizing conspiracy theories. Government officials, medical professionals, researchers, and any other authority figures who perpetrated and/or caused the persecution of a group, intentionally or not, should be publicly held accountable. Covering up past and present faults only risks amplifying the secrecy element of conspiracy theories and reinforcing their endorsement and generalization. The other key element, transparency, has been pointed by multiple authors as a critical principle with which to manage public health concerns, including epidemics, which are events of tremendous uncertainty (O’Malley et al., 2009; Carlsen and Glenton, 2016). Governments holding back information increase the risk that people “collectively generate alternative narratives” (Kou et al., 2017). Carlsen and Glenton (2016) reported similar conclusions and highlighted the importance of being transparent regarding the rationale of strategies used and the reasons for the decisions taken. Based on their systematic review of qualitative studies, Carlsen and Glenton (2016) believe that “transparency regarding uncertainty” would lead to “compliance with public health strategies.”

## Discussion

The present article asserted that conspiracy theories are narratives that can lead to violent radicalization *via* the thwarting of the universal needs of autonomy, competence, and relatedness. There is a consensus among experts that mistrust toward government representatives and authorities is central to the spreading of conspiracy theories during epidemics. Such mistrust toward authorities and influential figures could reflect the repetitive thwarting of needs. Accordingly, initiatives promoting community engagement are considered essential to ensure compliance with health measures during epidemics and to reduce the prevalence of endorsement of conspiracy theories; however, the best approach to regain trust is unknown (Vinck et al., 2019). The initiatives presented in this article aim to increase community engagement and trust toward authorities by satisfying the needs for autonomy, competence, and relatedness; however, they do require a certain basic level of trust from the population, which is a limitation to their potential efficacy. It is therefore not possible at the moment to determine the level of efficacy of the presented initiatives. To try to ensure their efficacy, governments will have to multiply their voices at various places simultaneously and deliver consistent messages and information. Governments could also collaborate with alternative and local leadership figures present in the population (e.g., athletes, influencers, and religious figures, Coltart et al., 2017; Vinck et al., 2019). We encourage that the implementation of the proposed initiatives is paired up with an assessment of their usefulness and efficacy.

Some governments have already implemented channels of communication through which people can raise their concerns and ask questions (e.g., public consultations with the population and government representative talks including question periods). However, the effectiveness of these channels of communication can be hindered, as some governments do not end up holding their end of the bargain and implement the changes promised. If governments do not listen to the concerns of their population, transparently communicate, and support the satisfaction of the needs of the population for autonomy, competence, and relatedness, one unavoidable consequence is that the endorsement of conspiracy theories will increase and, therefore, negatively affect the public health of their population.

## Data Availability Statement

The original contributions presented in the study are included in the article/supplementary material, further inquiries can be directed to the corresponding author.

## Author Contributions

M-JL conceptualized the manuscript and conducted the literature search, wrote the original draft, contributed to the critical review of the manuscript, and edited the manuscript. FP supervised the conceptualization of the manuscript and contributed to the critical review of the manuscript. Both authors contributed to the article and approved the submitted version.

## Conflict of Interest

The authors declare that the research was conducted in the absence of any commercial or financial relationships that could be construed as a potential conflict of interest.

## Publisher’s Note

All claims expressed in this article are solely those of the authors and do not necessarily represent those of their affiliated organizations, or those of the publisher, the editors and the reviewers. Any product that may be evaluated in this article, or claim that may be made by its manufacturer, is not guaranteed or endorsed by the publisher.

## Footnotes

1
https://www.sciencepresse.qc.ca/covid-19
