# Corrigendum: Conspiracy Theories: A Public Health Concern and How to Address It

**DOI:** 10.3389/fpsyg.2021.748874

**Published:** 2021-09-27

**Authors:** Marie-Jeanne Leonard, Frederick L. Philippe

**Affiliations:** Department of Psychology, Université du Québec à Montréal, Montreal, QC, Canada

**Keywords:** conspiracy theories, public health, self-determination theory, needs, radicalization, conspiracy beliefs

In the original article, there was an error in the Funding statement as published. The correct grant number for Social Sciences and Humanities Research Council of Canada is #430-2018-00740. The corrected Funding statement is shown below.

## Funding

This work was supported by the Social Sciences and Humanities Research Council of Canada (Grant number #430-2018-00740).

In the original article, there were also errors in the text. Part of a sentence was not adequately corrected, and words were not italicized and/or the first letter of the words were not capitalized.

A correction has been made to the section *Introduction, Paragraph 2*. The corrected paragraph is shown below.

We argue that conspiracy theories should be considered as narratives that can lead to violent radicalization and, as such, this phenomenon represents an important public health issue. Conspiracy theories are better understood *via* the 3N model of radicalization (Kruglanski et al., 2019) and self-determination theory (Ryan and Deci, 2017). The 3N model specifies three pillars in the radicalization process that align with the understanding of conspiracy theories (i.e., *Need, Narrative, and Network*), while self-determination theory deepens the understanding of the *Need* pillar.

Similarly, a correction has also been made to *Conspiracy Theories, Paragraph 3*. The corrected paragraph is shown below.

When one adheres to a narrative, they then seek the presence of like-minded individuals, forming a *Network* (Kruglanski et al., 2019). In the past few months, those who endorsed a conspiracy theory on SARS-CoV-2 connected *via* social media, creating echo chambers. These echo chambers spread the reinforcement of both individual and collective actions that exacerbated tensions between civilians and impeded the initiatives of authorities to halt the propagation of the virus around the globe, propelling actions such as civil disobedience, maskless demonstrations, or a refusal to get tested and vaccinated.

Additionally, part of a sentence was not adequately corrected and was repeated twice. “thus exacerbating the current thus exacerbating the thwarted needs related…”

A correction has been made to the section *Conspiracy Theories, Paragraph 1*. The corrected paragraph is shown below.

*Need* refers to the motivation to recover significance following its loss due to an adverse event (Kruglanski et al., 2019). Specifically, this significance loss can be conceptualized as the thwarting of three psychological needs that were found to be universal: competence, autonomy, and relatedness (Ryan and Deci, 2017). Indeed, the satisfaction or frustration of these three psychological needs influences how people perceive and react to an event (Ryan and Deci, 2017). The satisfaction of these psychological needs is considered as a continual quest, and when they are not satisfied or are frustrated, people naturally seek to fulfill them (Sheldon and Gunz, 2009; Ryan and Deci, 2017). They are, therefore, considered as core determinants of motivation that lead one to act on their environment and to carry certain objectives (Sheldon and Gunz, 2009; Ryan and Deci, 2017). Conspiracy theories unfold following an important event that hinders the perception of control of an individual (autonomy), the ability of an individual to make sense of the world (competence), and connectedness of an individual (relatedness; Ryan and Deci, 2017; van Prooijen, 2020). In 2020, many countries enforced a lockdown for months, a significant event that precipitated economic uncertainty and restrained individual freedom. Many have perceived their basic needs as thwarted as they lost control over their usual occupations, they were cut off from their loved ones, and authorities disseminated mixed messages because they did not (and still do not) fully understand the new virus. Such stressful events are likely to reactivate the recall of past personal life events that were need thwarting in a similar fashion (e.g., experiences of ostracism, natural disasters, or other traumas), thus exacerbating the current need thwarting experience of the pandemic (Philippe and Houle, 2020). A vulnerability to the reactivation of such need thwarting memories can motivate one to retrieve significance by finding compensatory ways to fulfill those needs, making one cognitively receptive to new narratives.

Lastly, an in-text citation was written incorrectly. Instead of “as time goes by Carlsen and Glenton (2016).” it should be “as time goes by (Carlsen and Glenton, 2016).”

A correction has been made to the section *Potential Initiatives, Paragraph 1*. The corrected paragraph is shown below.

Policymakers and authorities should be careful to not circulate mixed and confusing messages at a given time (Abramowitz et al., 2017), as past epidemics were marked by the dissemination of ambivalent messages on the virus at play (Kou et al., 2017). However, changing information is not necessarily synonymous with mixed messages (Carlsen and Glenton, 2016). To prevent their information and messages from being considered as mixed, policymakers and authorities should “acknowledge” the presence of uncertainty and that the information disseminated will be adjusted as time goes by (Carlsen and Glenton, 2016). Otherwise, people might consider and interpret mainstream information as misinformation (Ball and Maxmen, 2020). Furthermore, Ball and Maxmen (2020) emphasized that authorities and policymakers should describe the reasons and rationale that “guide” the changed decisions during an epidemic.

The authors apologize for these errors and state that they do not change the scientific conclusions of the article in any way. The original article has been updated.

## Publisher's Note

All claims expressed in this article are solely those of the authors and do not necessarily represent those of their affiliated organizations, or those of the publisher, the editors and the reviewers. Any product that may be evaluated in this article, or claim that may be made by its manufacturer, is not guaranteed or endorsed by the publisher.

## References

[B1] Abramowitz S. McKune S. L. Fallah M. Monger J. Tehoungue K. Omidian P. A. (2017). The opposite of denial: social learning at the onset of the Ebola emergency in Liberia. J. Health Commun. 22, 59–65. 10.1080/10810730.2016.1209599 28854129

[B2] Ball P. Maxmen A. (2020). Battling the infodemic. Nature 581, 371–374. 32461658 10.1038/d41586-020-01452-z

[B3] Carlsen B. Glenton C. (2016). The swine flu vaccine, public attitudes, and researcher interpretations: a systematic review of qualitative research. BMC Health Serv. Res. 16:203. 27338141 10.1186/s12913-016-1466-7 PMC4919843

[B4] Kou Y. Gui X. Chen Y. Pine K. H. (2017). Conspiracy talk on social media: collective sensemaking during a public health crisis. Proc. ACM Hum. Comput. Interact. 1:61.

[B5] Kruglanski A. W. Bélanger J. J. Gunaratna R. (2019). The Three Pillars of Radicalization: Needs, Narratives, and Networks. New York: Oxford University Press.

[B6] Philippe F. L. Houle I. (2020). Cognitive integration of personal or public events affects mental health: investigating memory networks in a case of natural flooding disaster. J. Pers. 88, 861–873. 10.1111/jopy.12531 31808166

[B7] Ryan R. M. Deci E. (2017). Self-Determination Theory: Basic Psychological Needs in Motivation, Development, and Wellness. New York: Guilford Press.

[B8] Sheldon K. M. Gunz A. (2009). Psychological needs as basic motives, not just experiential requirements. J. Pers. 77, 1467–1492. 10.1111/j.1467-6494.2009.00589.x 19678877

[B9] van Prooijen J. W. (2020). An existential threat model of conspiracy theories. Eur. Psychol. 25, 16–25. 10.1027/1016-9040/a000381

